# Supplementary material for: Concurrent warming, freshening and cessation of deep convection in the Labrador Sea raised its sea level to a record high
Source: Nat Commun. 2025 Nov 28;16:10721. doi: 10.1038/s41467-025-65747-3 (PMC12663177; doi:10.1038/s41467-025-65747-3)
Supplement: Supplementary file 1 — Supplementary Information [file 41467_2025_65747_MOESM1_ESM.pdf]

*Supplementary Information for*

# Concurrent warming, freshening and cessation of deep convection in the Labrador Sea raised its sea level to a record high

**Igor Yashayev[1], Yang Zhang[2,3]**

[1]Bedford Institute of Oceanography, Dartmouth, N.S., Canada
[2]School of Marine Science and Policy, University of Delaware, Lewes, Delaware, USA
[3]Scripps Institution of Oceanography, University of California, San Diego, La Jolla, CA, USA

*Corresponding author:* Igor Yashayev

*Emails:* Igor.Yashayev@dfo-mpo.gc.ca; Labrador.Sea@gmail.com

*Phone:* (782)-640-9437

*Table of Content:*

***Supplementary Figure 1.*** 1948–2025 central Labrador Sea full-depth temperature, salinity and density profiles **3**

***Supplementary Figure 2.*** 1948–2025 central Labrador Sea ocean state and atmospheric forcing metrics **4**

***Supplementary Note 1.*** Ocean state reporting with advanced oceanographic section plots **5**

***Supplementary Figure 3.*** 2015, 2016, 2018, 2023, 2024 and 2025 temperature, salinity and density sections based on the Labrador Sea repeat hydrography line AR7W observations **6**

***Supplementary Figure 4.*** 2018–2025 post-convective, April-to-August, composite temperature, salinity and density sections **8**

***Supplementary Figure 5.*** Developments and decays of Labrador Sea pycnostads **10**

***Supplementary Figure 6.*** Seasonal cycle of the detrended gridded and along-track altimetry-based sea level in the central Labrador Sea **11**

**Supplementary Figure 7.** Gridded and along-track altimetry-based sea level anomalies in the central Labrador Sea **12**

**Supplementary Figure 8.** Seasonal cycle of the total steric, thermosteric and halosteric heights **13**

**Supplementary Figure 9.** 1992–2025 sea level budget variability with seasonal cycle **14**

**Supplementary Figure 10.** 1990–2025 total steric, thermosteric and halosteric heights with seasonal cycle **15**

**Supplementary Figure 11.** 1992–2025 sea level budget variability with total steric, thermosteric and halosteric heights for the 10-1900 dbar layer **16**

**Supplementary Figure 12.** 1990–2025 central Labrador Sea steric, thermosteric and halosteric heights for the 10-1900 dbar layer **17**

***Supplementary Note 2.*** Reconstruction of thermosteric height changes from surface heat flux data **18**

**Supplementary Figure 13.** Examples of the multistep thermosteric height reconstruction optimization process **19**

**Supplementary Figure 14.** 1992–2025 sea level budget variability with detrended CSR GRACE data **20**

***Supplementary Table 1.*** Labrador Sea hydrographic observations collected by the Bedford Institute of Oceanography collected by the Bedford Institute of Oceanography in compliance with the Deep-Ocean Observation and Research Synthesis (DOORS) and World Ocean Circulation Experiment (WOCE) quality requirements **21**

***Supplementary Note 3.*** Responses to internal reviewers **22**

***Supplementary References*** **26**

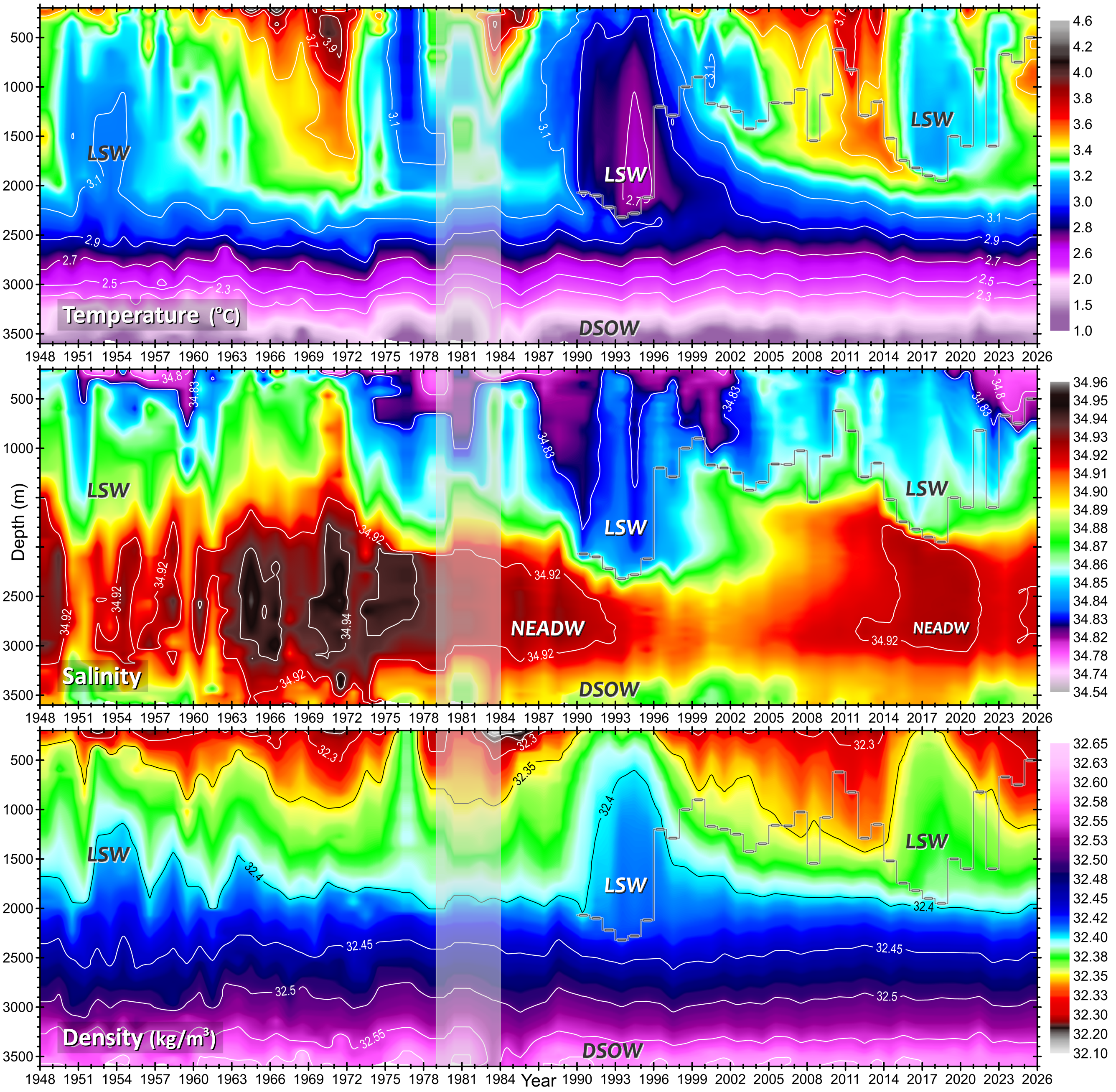


**Supplementary Figure 1.** *Top-down:* The 1948–2025 central Labrador Sea (CLS) yearly-averaged temperature, salinity and density profiles constructed from the quality-controlled, edited and calibrated ship (for all tears except for 2017 and 2021) and Argo float (for 2017 and 2021) observations. LSW, NEADW, and DSOW indicate Labrador Sea Water, Northeast Atlantic Deep Water, and Denmark Strait Overflow Water, respectively.
*Horizontal lines* indicate the convection depths computed (following Yashayaev, 2024) for the period covered with sufficient high-resolution hydrographic observations (1987–2025).

High-quality full-depth hydrographic observations collected between 1990 and 2019 (Supplementary Table 1) reveal weak changes in NEADW, suggesting that the sporadic salinity jumps 0.02 or larger before 1975 are likely due to instrumental issues (e.g., salinity standards and calibrations), whereas, during 1990–2019, exclusively, the accuracy of salinity was maintained at 0.0015 or higher.

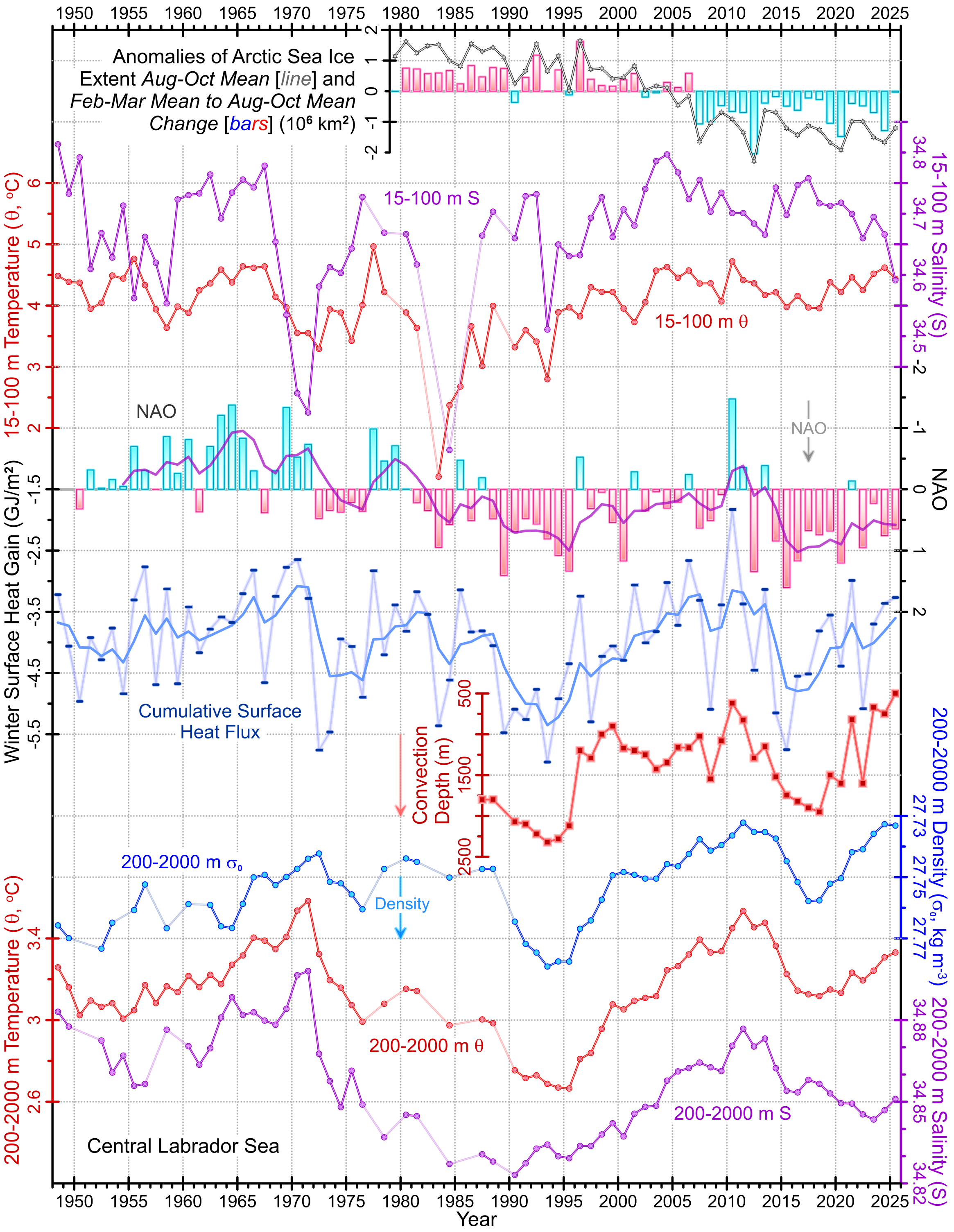


**Supplementary Figure 2.** *Top-down*: The Aug-Oct Arctic sea ice extent, and Feb-Mar to Aug-Oct extent change anomalies; 1948–2025 central Labrador Sea (CLS) 15-100 m vertically and annually averaged de-seasoned salinity ($S$) and temperature ($\theta$); *inverted* winter NAO index; CLS surface heat flux integrated over individually defined cooling seasons (*blue*); low-pass filtered, with left-side triangularly-weighted window, NAO and surface heat flux (*solid lines*); convection depth; CLS 200-2000 m vertically and annually averaged density ($\sigma_0$, referenced to 0 dbar), $\theta$ and S.

## Supplementary Note 1. Ocean state reporting with advanced oceanographic section plots

Supplementary Figs. 3 and 4 showcase, and allow to compare, the method used in our previous reporting of oceanographic conditions in the Labrador Sea (e.g., *Yashayaev, 2007; Yashayaev et al., 2007a, b*), and the innovative approach that we currently use to representing the state of the ocean on an arbitrary, here cross-basin, transect (e.g., *Yashayaev, 2024*).

The section plots based on the observations collected on the Atlantic Repeat 7-West (AR7W) hydrography line annually during a random week of May (June or July) were extensively used in the past assessments of the Labrador Sea oceanographic conditions (*e.g., Dickson et al., 2002; Lazier et al., 2002; Yashayaev, 2007; Yashayaev et al., 2007a, b*). Supplementary Figs. 3 shows six examples of vertical sections, each comprising a fairly limited number (25-40) of ship-based profiles. The 2015, 2016 and 2018 temperature and salinity profiles were properly calibrated by the author to 0.0015 and 0.0010°, respectively; while the 2023, 2024 and 2025 profiles were adjusted to data from other cruises for the lack of high quality water sample salinity measurements. Other examples of AR7W sections can be found our previous study (*Yashayaev, 2024*, Supplementary Fig. 4).

There are certain limitation in using infrequent ship-based observations.
Any analysis of such data is limited to the depth level below 200 m, as the top 200 m layer is dominated by regular (climatological) and irregular (of varying magnitude and timing) seasonality. Until the year-round Argo float data became integrated into the state of the ocean assessment process, the ship surveys were the only means of gathering information about the deep-sea state.

Also, as evident from the presented sections, and Fig. 2 of the "*Results*" section, the use of one-time annual, especially spring, surveys, does not provide adequate and sufficient annual representation of the top 1000 m layer state, including the convection depth, as all layers in this depth range, including the winter mixed layer, undergo significant transformations. If in the years with relatively deep winter convection (e.g., 2015–2018), the convection depth can be crudely estimated from the ship-based sections, but once convection becomes weak (e.g., 2023–2025), its depth cannot be determined from the spring and later ship-based observations without a large error.

An alternative approach to oceanographic section plotting, presently used to provide both accurate assessment of the state of the entire water column across the sea and estimate the depth of the past winter mixing, was developed and implemented by the author (*Yashayaev, 2024*).
Supplementary Fig 4 shows composite annual sections built basing on this approach.

These oceanographic sections are constructed following the approach earlier developed and implemented by the author (*Yashayaev, 2024*). The underlying principles include: [1] using all quality checked, edited and calibrated oceanographic data collected by all profiling platforms (ships and Argo floats, prospectively sea gliders) within a certain distance of an arbitrarily chosen section; [2] restricting "sampling" period to similar oceanographic conditions (here, after winter convection and before fall); [3] projecting selected observation onto the chosen section in the *hybrid coordinate system*, combining the advantages of the bathymetry-based and shortest distance based approaches to spatial data interpolation (*Yashayaev, 2024*).

Note clear representation of annual hydrographic conditions, e.g., the inflow of the fresher Arctic and saltier Atlantic waters from the Labrador (left) and Greenland (right) shelves, respectively.

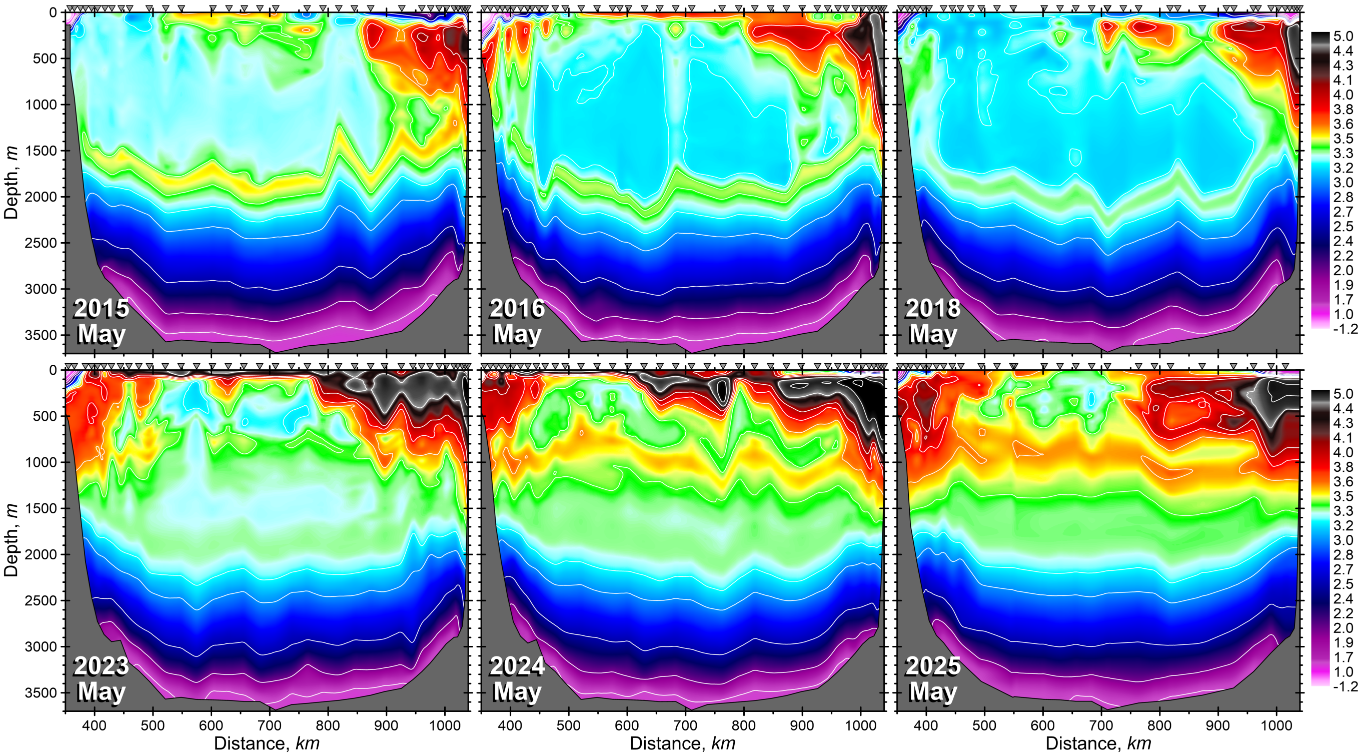


**Supplementary Figure 3a.** Misery Point (Labrador) to Cape Desolation (Greenland) full-depth potential temperature (°C) sections constructed from the shipboard observations collected during short May periods of 2015, 2016, 2018, 2023, 2024 and 2025 (shelves are excluded).

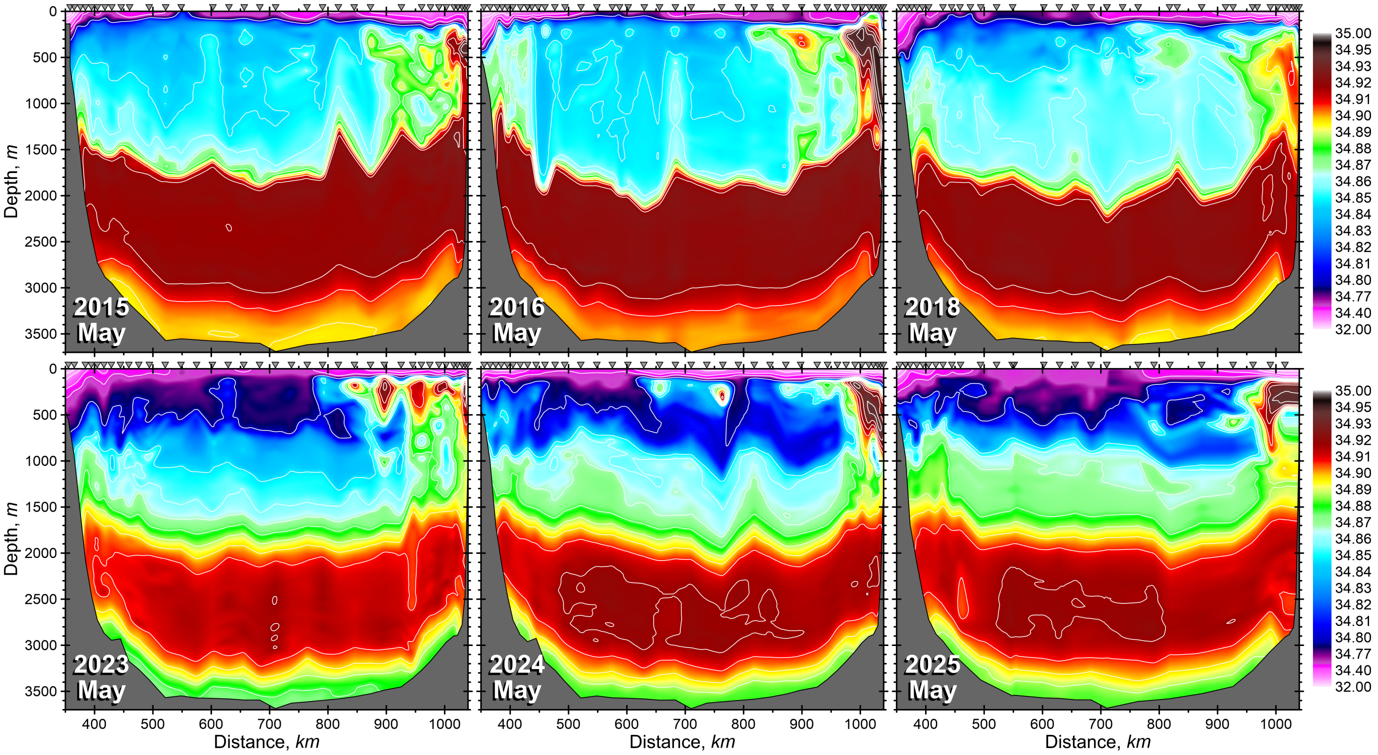


**Supplementary Figure 3b.** Misery Point (Labrador) to Cape Desolation (Greenland) full-depth salinity sections constructed from the shipboard observations collected during short May periods of 2015, 2016, 2018, 2023, 2024 and 2025 (shelves are excluded).

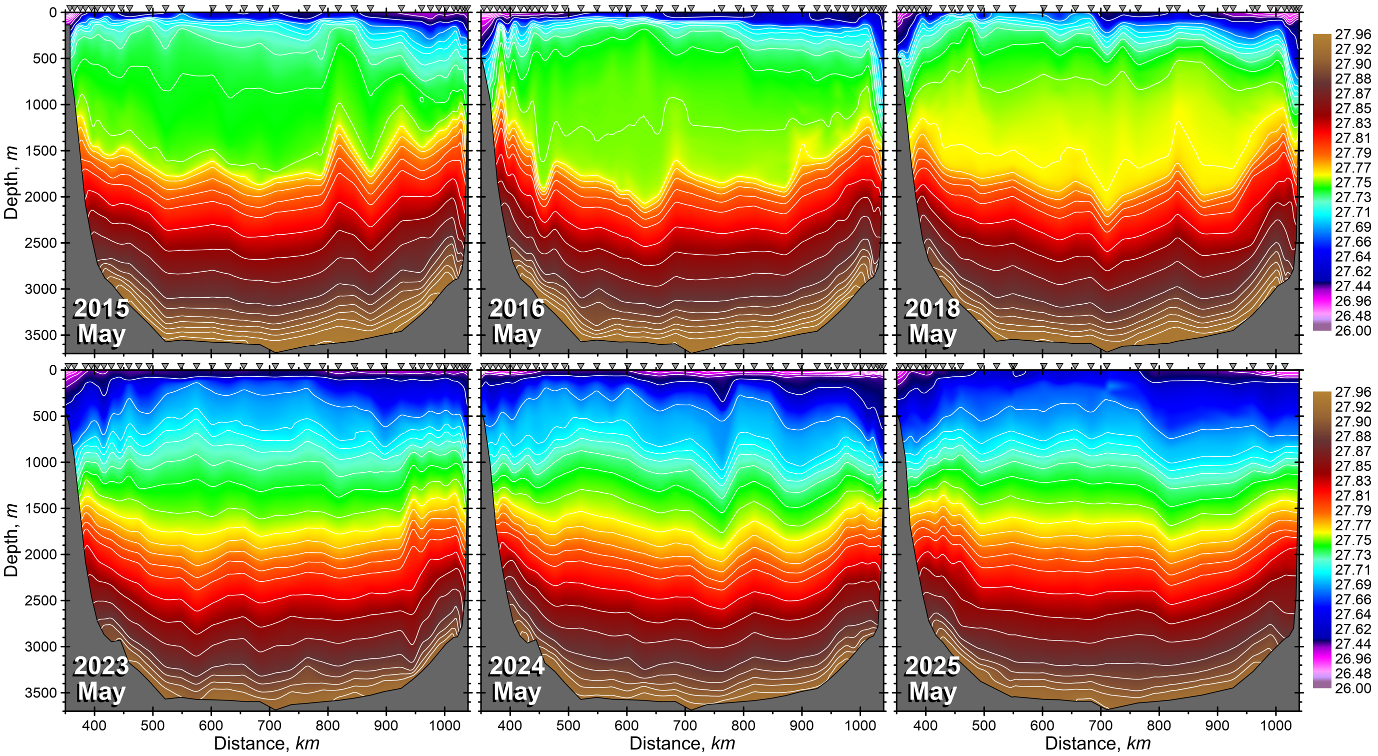


**Supplementary Figure 3c.** Misery Point (Labrador) to Cape Desolation (Greenland) full-depth potential density (kg/m$^3$) sections constructed from the shipboard observations collected during short May periods of 2015, 2016, 2018, 2023, 2024 and 2025 (shelves are excluded).

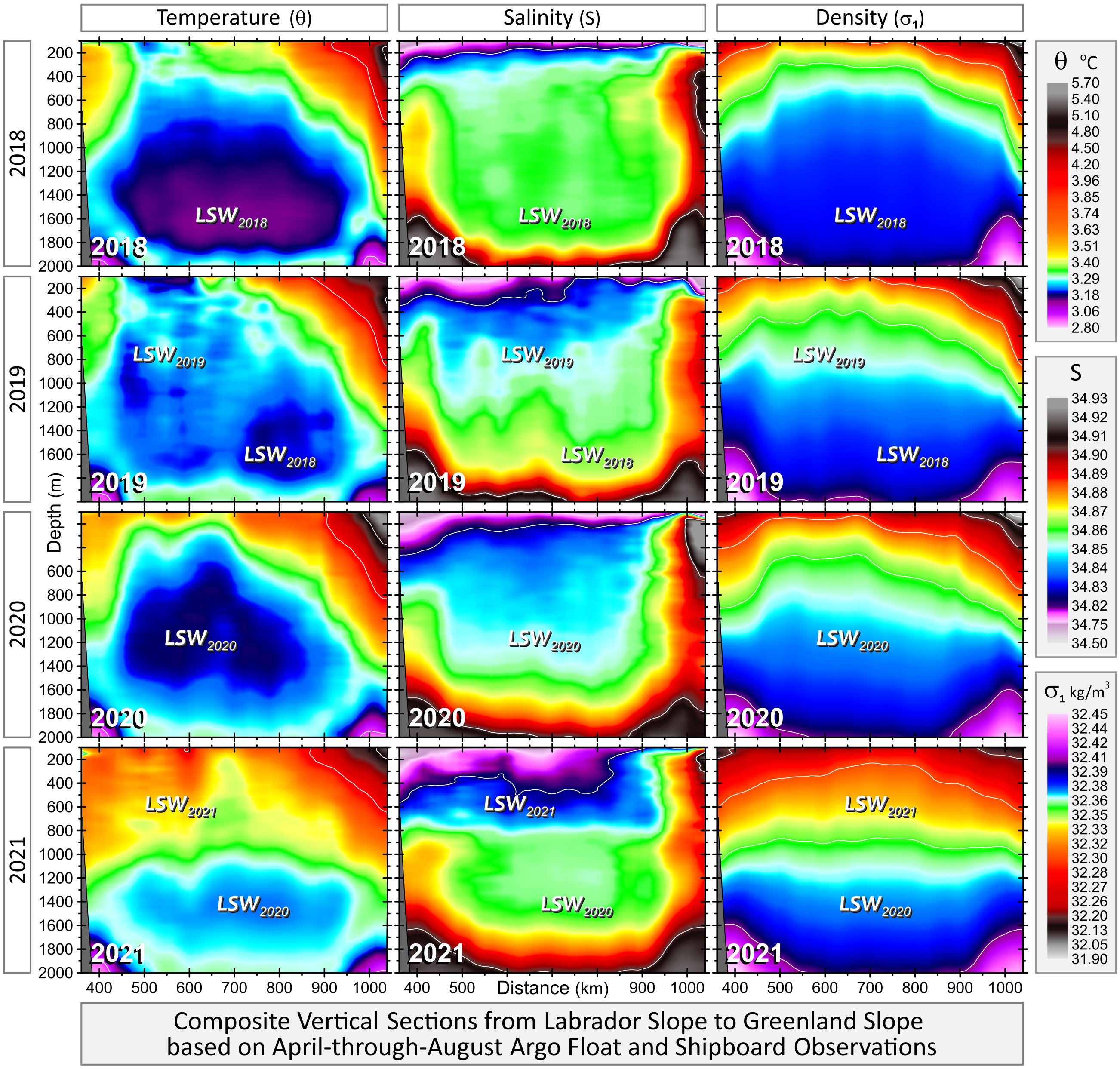


**Supplementary Figure 4a.** Labrador Sea post-convective, April-to-August, composite temperature (°C), salinity and density (kg/m$^3$) sections constructed for the years from 2018 to 2021.

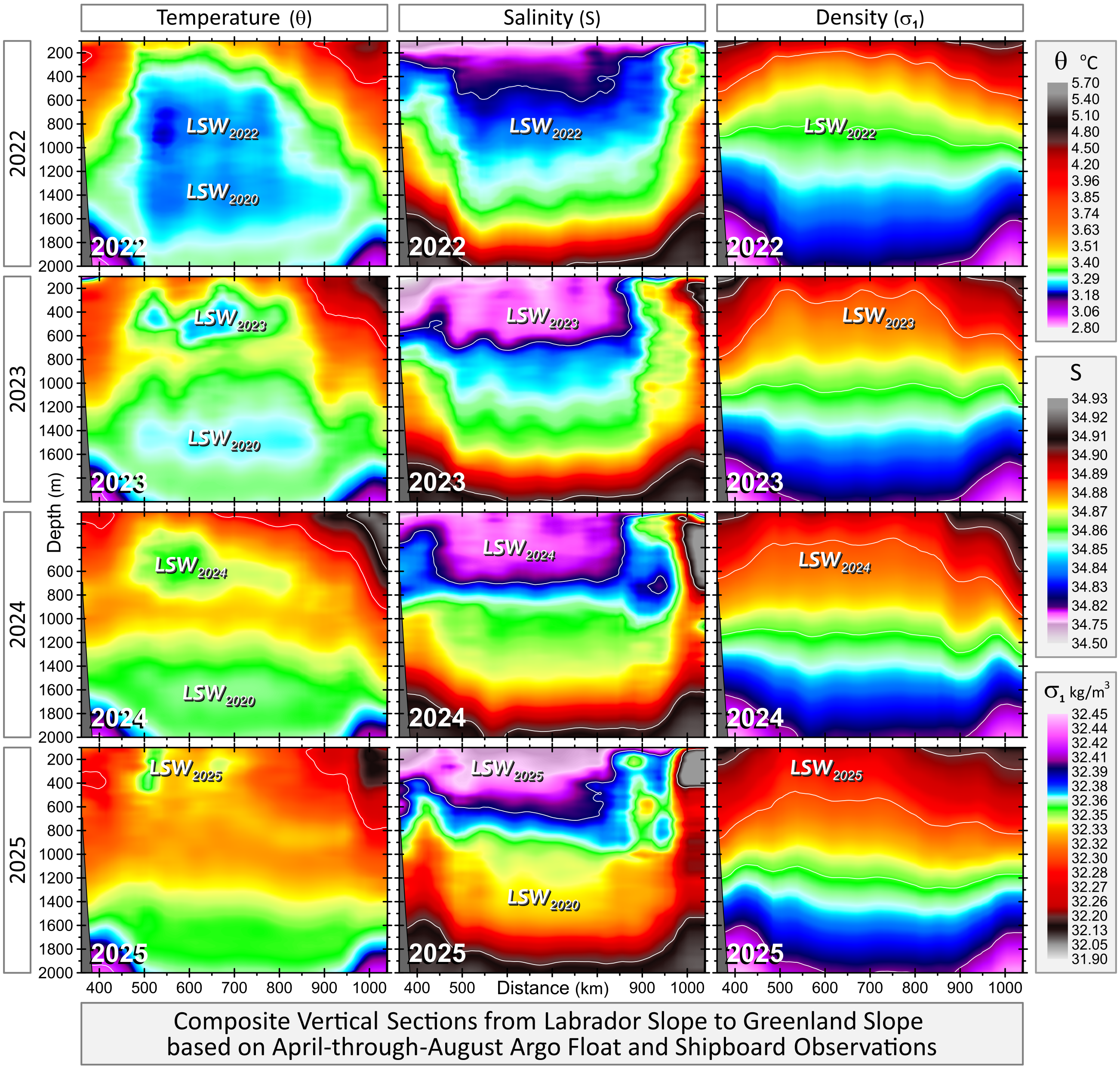


**Supplementary Figure 4b.** Labrador Sea post-convective, April-to-August, composite temperature (°C), salinity and density (kg/m$^3$) sections constructed for the years from 2022 to 2025.

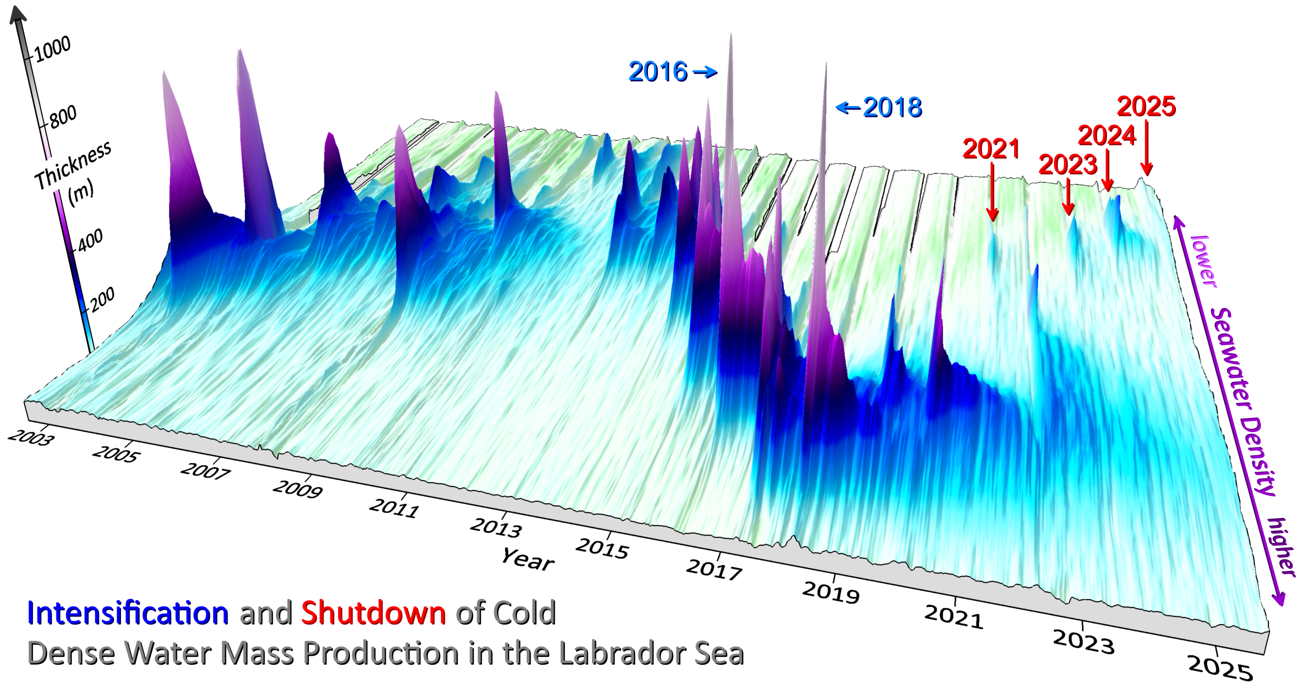


**Supplementary Figure 5.** The thickness of overlapping 0.005 kg/m$^3$-wide density layers in central Labrador Sea with weekly-to-biweekly temporal resolution between 2002 and 2025.

The view shown in this figure is based on the same values as the bottom panel of Fig. 2 in the "*Results*" section. The values underlying both figures are the quality-controlled and calibrated Argo float and ship-based observations.

The height of the peaks in this figure indicates the thickness of one or more concurrent pycnostads within the 10-2000 m layer of the CLS. The cases when more than one pycnostad concurrently coexist over the water column are often associated with a reduction of winter convection following a strong convection year or years. This situation is evident in the last five years of the record.

The blue and red numbers indicate the years of particularly thick and thin pycnostads, respectively.

The pycnostad development and prevalence cycles have direct impact on sea level.

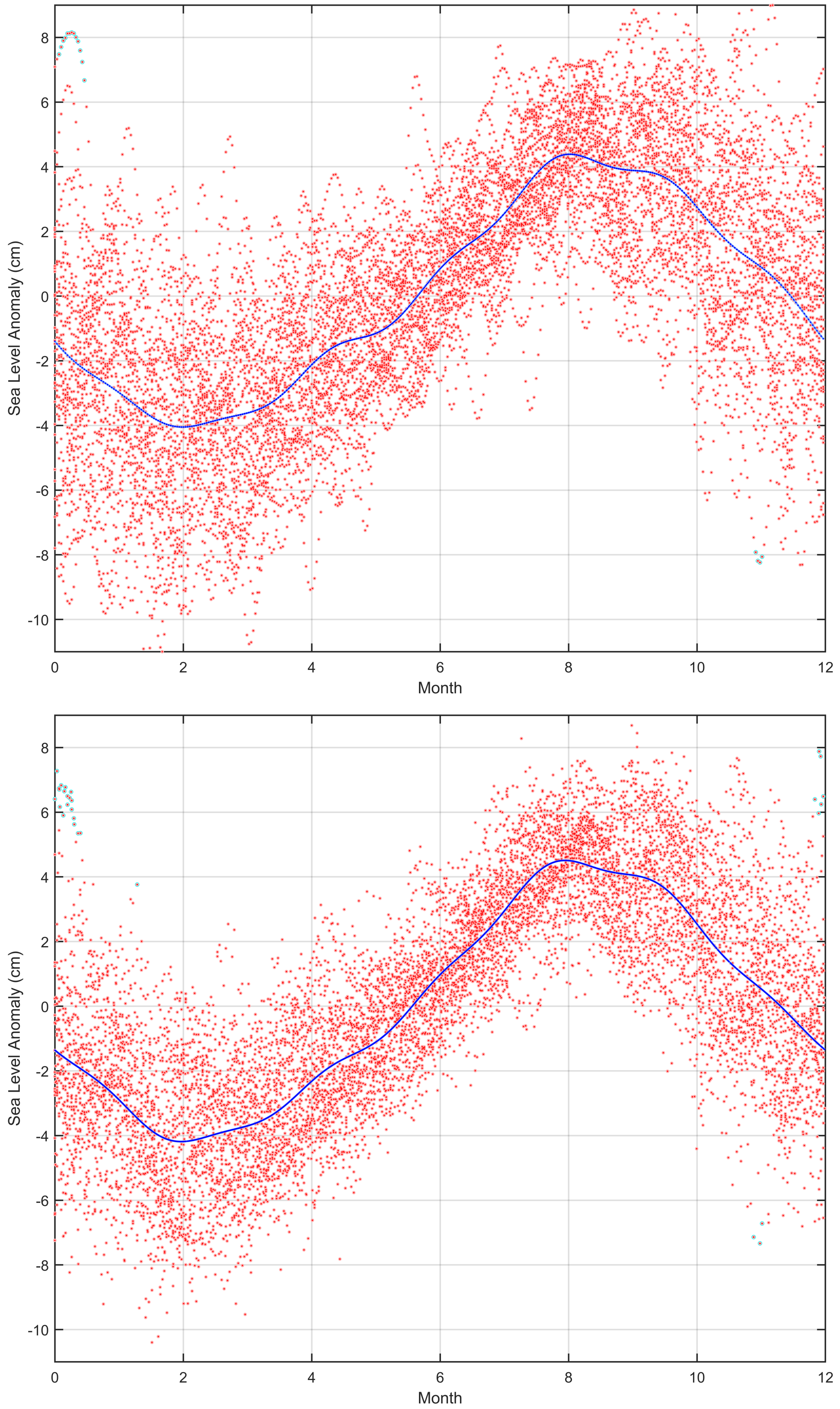


**Supplementary Figure 6.** Seasonal cycle of the gridded (*upper*) and along-track (*lower*) altimetry-based SLA in the CLS with the polynomial SLA anomaly trend removed from individual data values (*red dot clouds*). Blue lines represent the regular seasonal cycle.

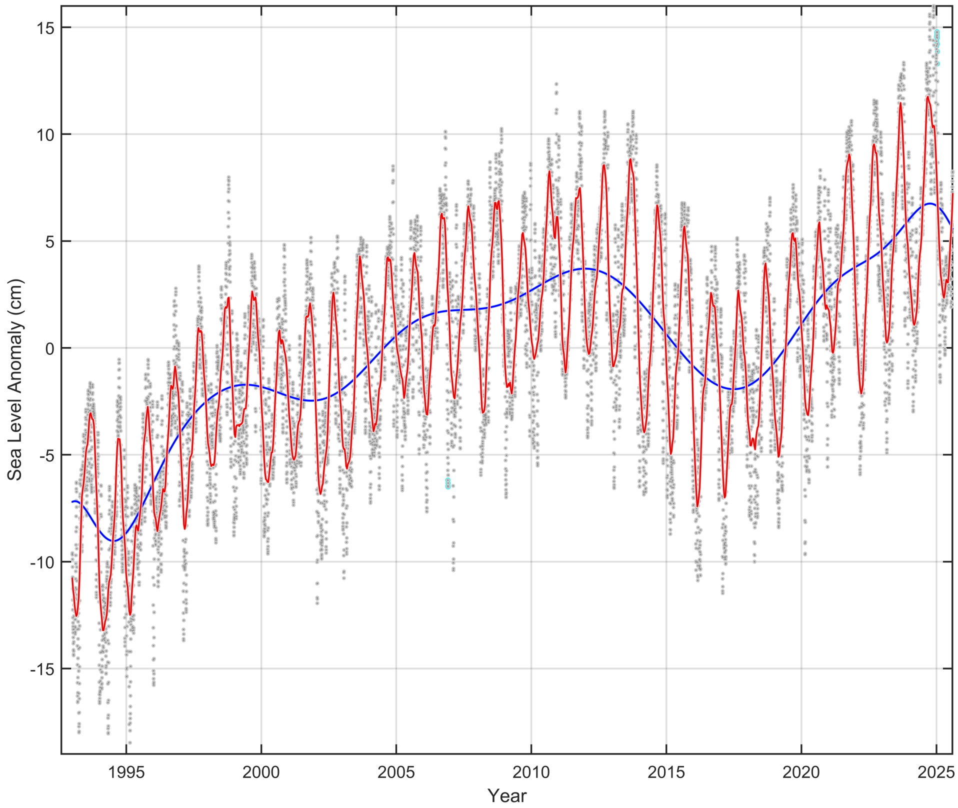


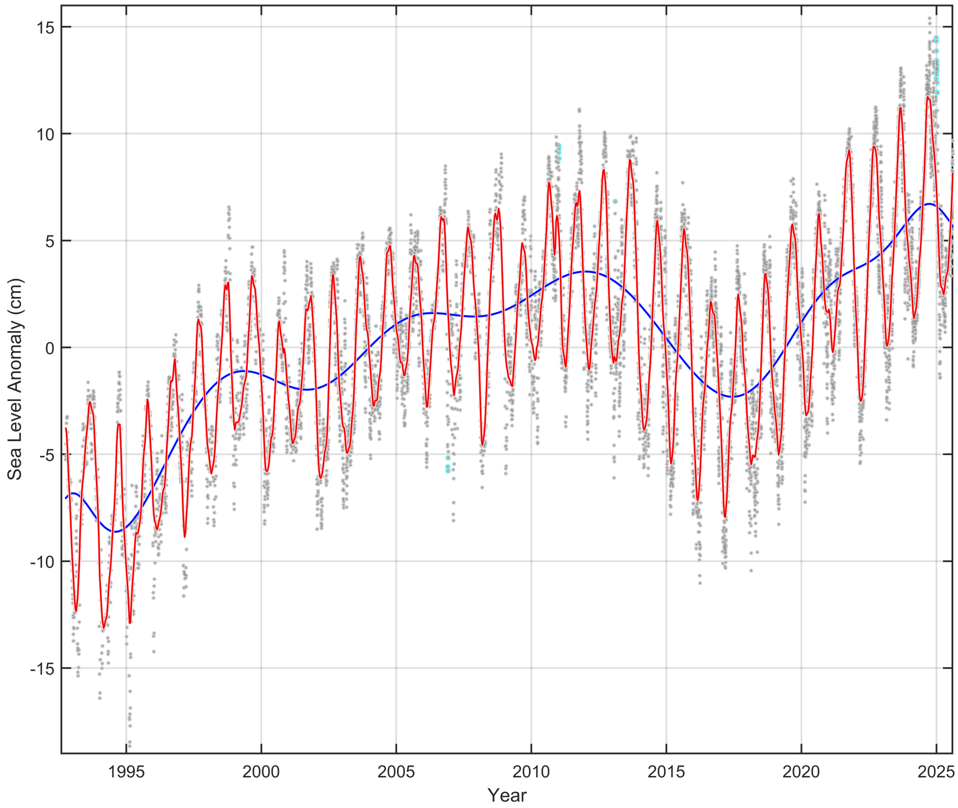


**Supplementary Figure 7.** Gridded (*upper*) and along-track (*lower*) altimetry-based sea level anomalies (SLA) in the central Labrador Sea (CLS). Blue and red lines represent polynomial fits of deseasoned SLA without and with the regular and irregular seasonal signals.

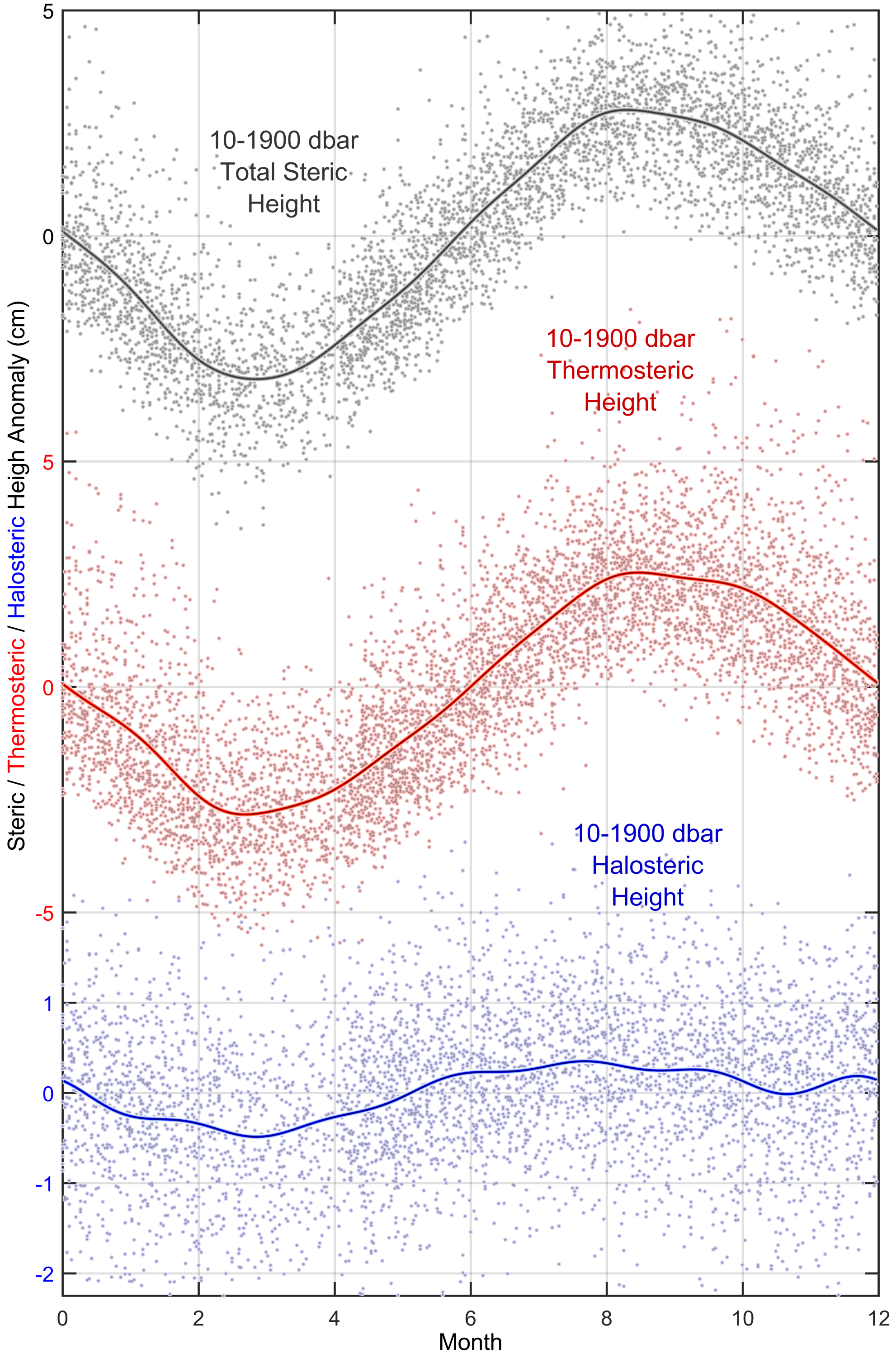


**Supplementary Figure 8.** CLS steric, thermosteric and halosteric height seasonality. *Top-down* (cm): the original upper (10-1900 dbar) layer steric (*grey/black*), thermosteric (*red*) and (*blue*) halosteric height observations with outliers and low-frequency signals removed (*dots*; the *dot clouds* capture seasonal and shorter-term variability); regular or climatological seasonal cycles (*lines*).

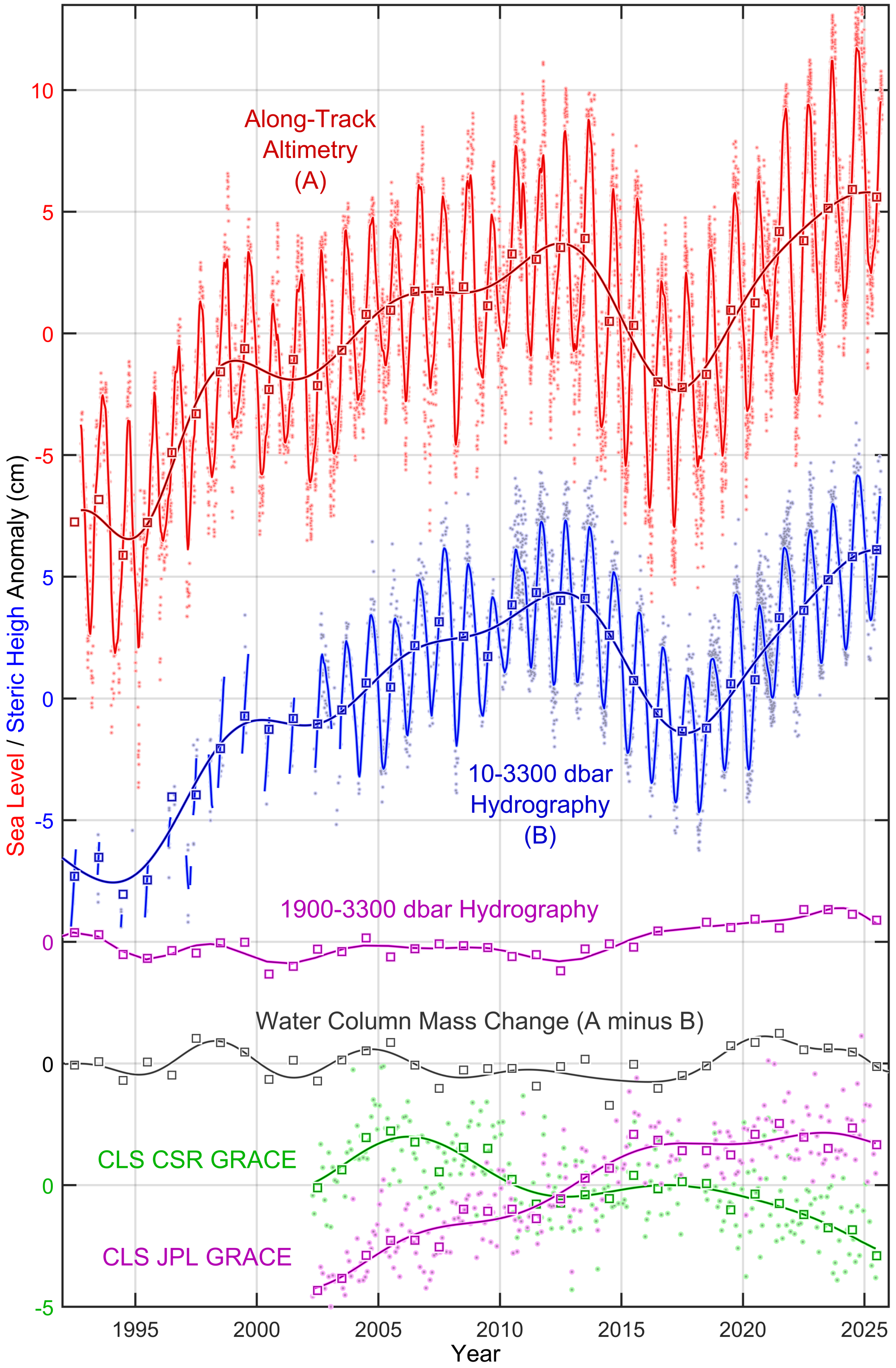


**Supplementary Figure 9.** 1992–2025 sea level budget variability in the central Labrador Sea. *Top-down* (cm): the along-track satellite altimetry-based sea level (*red*); full-depth (*blue*) and deep-layer (*purple*) steric heights; water column mass based on subtracting full-depth steric height from sea level (*grey/black*); and on CSR (*green*) and JPL (*purple*) GRACE data. Outliers have been removed from all original values. *Squares*, underlined with optimal fits, indicate yearly averaged deviations from the respective regular seasonal cycles. The summed regular seasonal, irregular seasonal and low-frequency signals of (A) and (B) are shown with *lighter lines*.

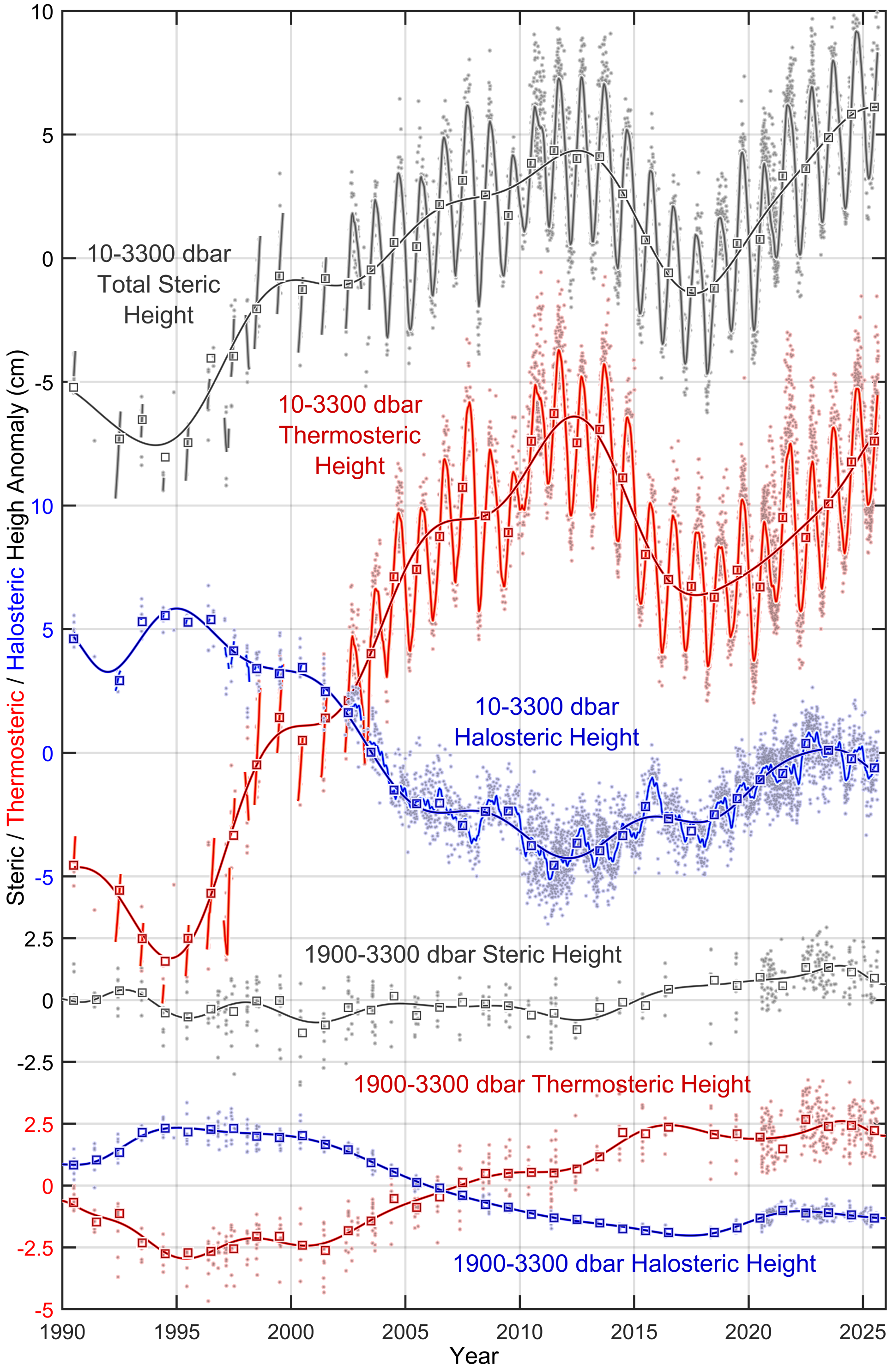


**Supplementary Figure 10.** 1990–2025 central Labrador Sea steric, thermosteric and halosteric heights. *Top-down* (cm): full-depth (10-3300 dbar) steric (*grey/black*), thermosteric (*red*) and halosteric (*blue*), and deep-layer (1900-3300 dbar) steric (*grey/black*), thermosteric (*red*) and halosteric (*blue*) heights. Outliers have been removed from all original values. *Squares*, underlined with optimal polynomial fits, indicate yearly averaged deviations from the respective regular seasonal cycles. The summed regular seasonal, irregular seasonal and low-frequency full-depth height signals are shown with *lighter lines*.

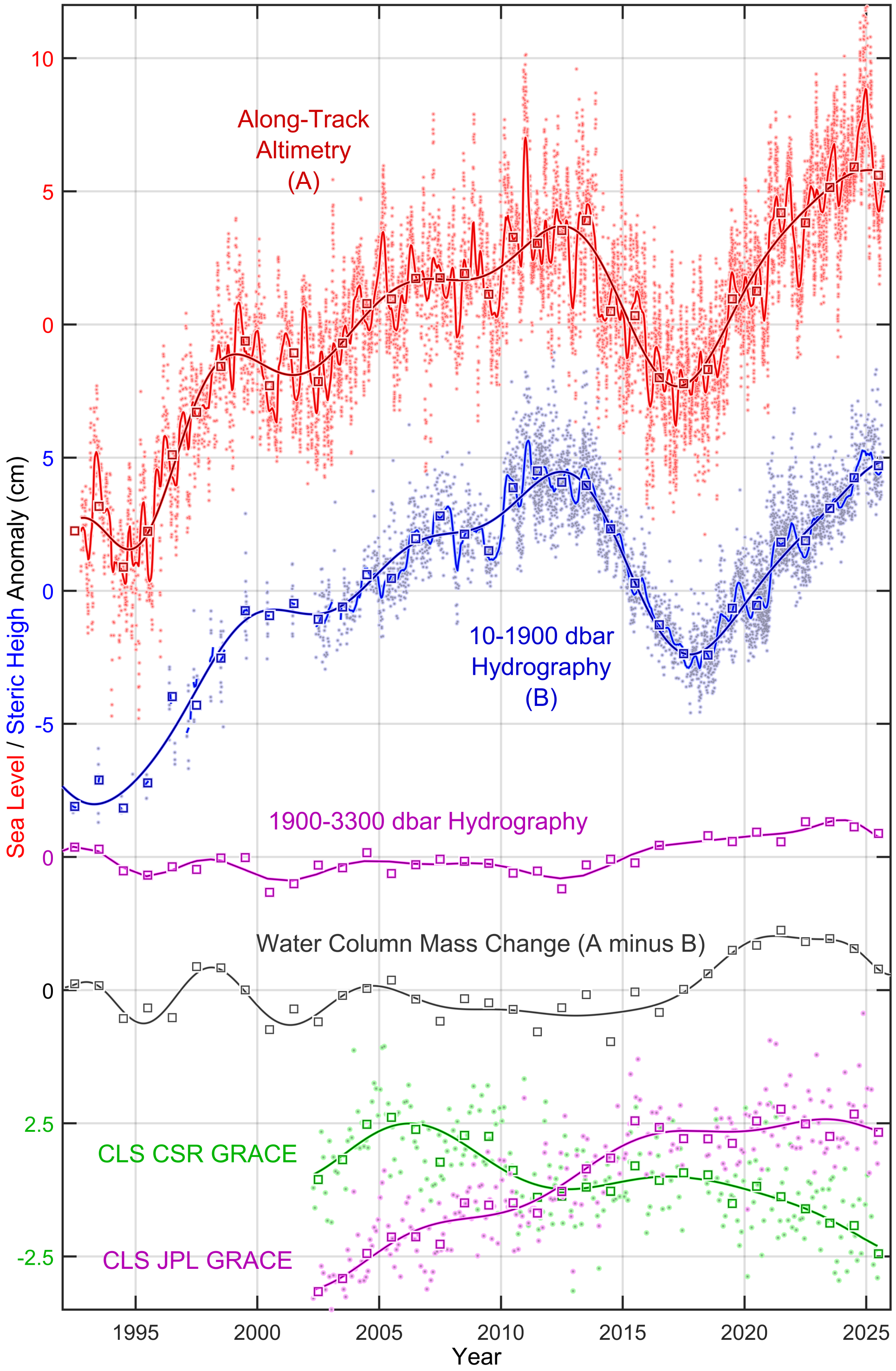


**Supplementary Figure 11.** 1992–2025 sea level budget variability in the central Labrador Sea. *Top-down* (cm): the along-track satellite altimetry-based sea level (*red*); upper (10-1900 dbar, *blue*) and deep (1900-3300 dbar, *purple*) layer steric heights; water column mass based on subtracting upper-layer steric height from sea level (*grey/black*); and on CSR (*green*) and JPL (*purple*) GRACE data. Outliers and respective regular (climatological) seasonal cycles have been removed from all original values. *Squares*, underlined with optimal fits, indicate the yearly averaged residuals. The summed irregular seasonal and low-frequency signals of (A) and (B) are shown with *lighter lines*.

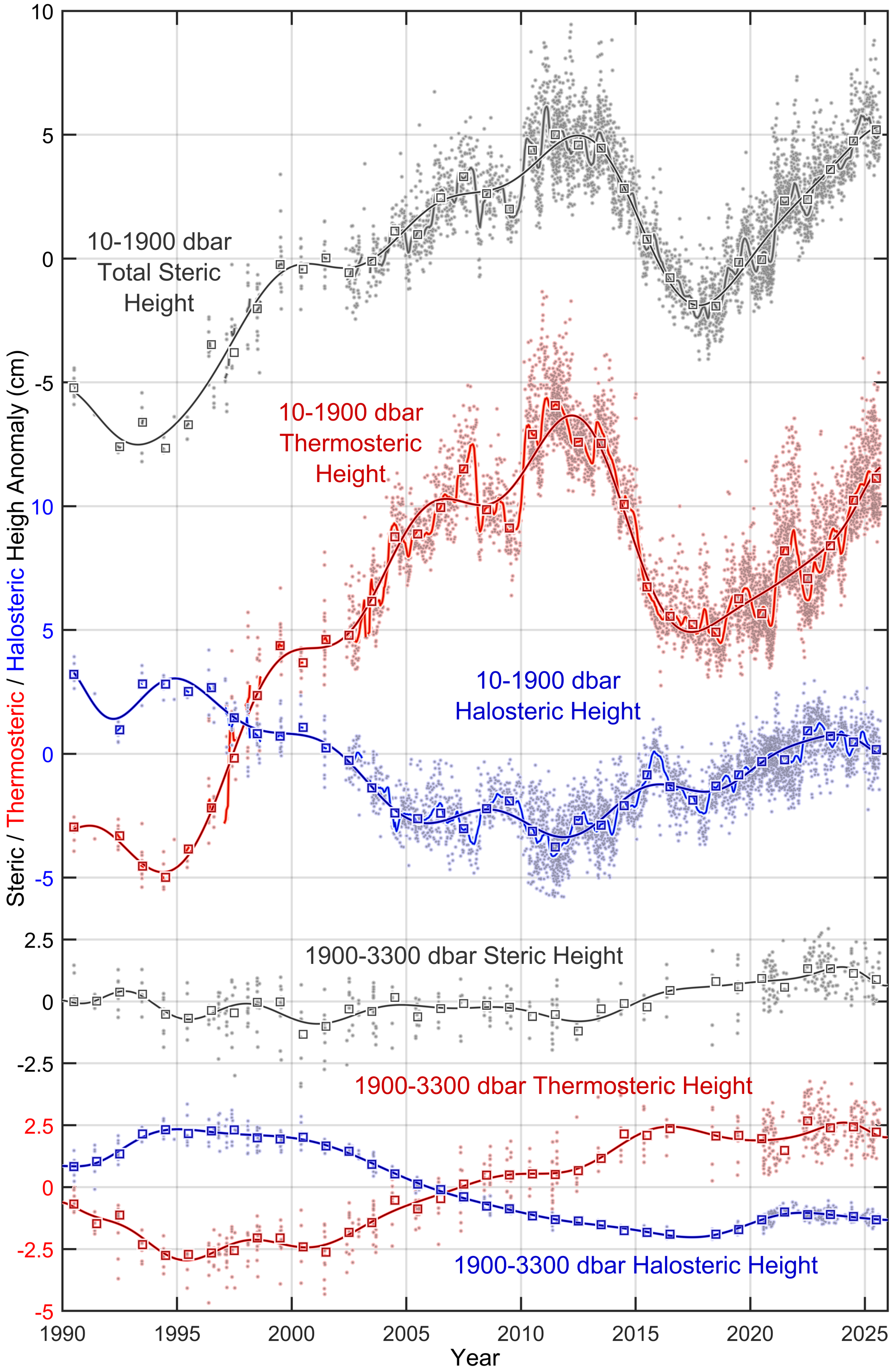


**Supplementary Figure 12.** 1990–2025 central Labrador Sea steric, thermosteric and halosteric heights. *Top-down* (cm): upper (10-1900 dbar) total steric (*grey/black*), thermosteric (*red*) and halosteric (*blue*), and deep (1900-3300 dbar) steric (*grey/black*), thermosteric (*red*) and halosteric (*blue*) heights. Outliers and respective regular (climatological) seasonal cycles have been removed from all original values. *Squares*, underlined with optimal polynomial fits, indicate the yearly averaged residuals. The summed irregular seasonal and low-frequency full-depth height signals are shown with *lighter lines*.

## Supplementary Note 2. Reconstruction of thermosteric height changes from surface heat flux data

Total *Winter Surface Heat Loss* (WSHL) and total *Summer Surface Heat Gain* (SSHG), or, alternatively to SSHG, mean *Summer Heat Peak* (SHP), have been low-pass filtered with independently-varying left-side triangular window sizes forming 625 window-size combinations. The low-pass filtered WSHL and SSHG|SHP have then been added together with SSHG|SHP multiplied by relative weight, labeled here as *Summer Heat Peak Scale Factor*.

Each of the resulting series is correlated with the yearly-averaged thermosteric heights to find the optimal configuration of the input variables. As examples of output from this optimization procedure, in Supplementary Figure 13, we show color-mapped correlation coefficients for 4, 5, 6 and 7 year-long WSHL left-side triangular filter windows. The corresponding correlation (coefficient of determination) peaks are indicated and labeled with the respective correlation and coefficient of determination values. Extensively repeated iterations reveal a distinct unique stable solution of the optimization problem. All tested combinations of WSHL and SSHG|SHP window sizes and *Summer Heat Peak Scale Factor* converge on a single and strong correlation high representing the best (closest) fit of the yearly-averaged thermosteric height series.

The highest correlation is achieved by combining 7 and 13 year low pass filtered WSHL and SHP/SSHG, respectively, after multiplying SHP by ~26. Please note that the results shown in this figure are for SHP. The optimizations with SSHG and SHP-SSHG hybrid yield similar results.

As explained in the main text, unlike SSHG, found by integrating Net Surface Heat Flux (NSHF) over entire warming periods of different duration, SHP is computed by averaging NSHF over fixed-length time intervals (e.g., 15, 21, 31 days) centered on annual NSHF highs (peaks). Switching between SSHG and SHP does not really alter the result of the thermosteric reconstructive optimization, although SHP gives slightly higher correlations than SSHG.
We assume that even better results can be achieved by adding SSHG and SHP together with different weights and, particularly, by using different spatial domains for computing SSHG and WSHL as SSHG has a much broader spatial signature than WSHL according to our extended results.

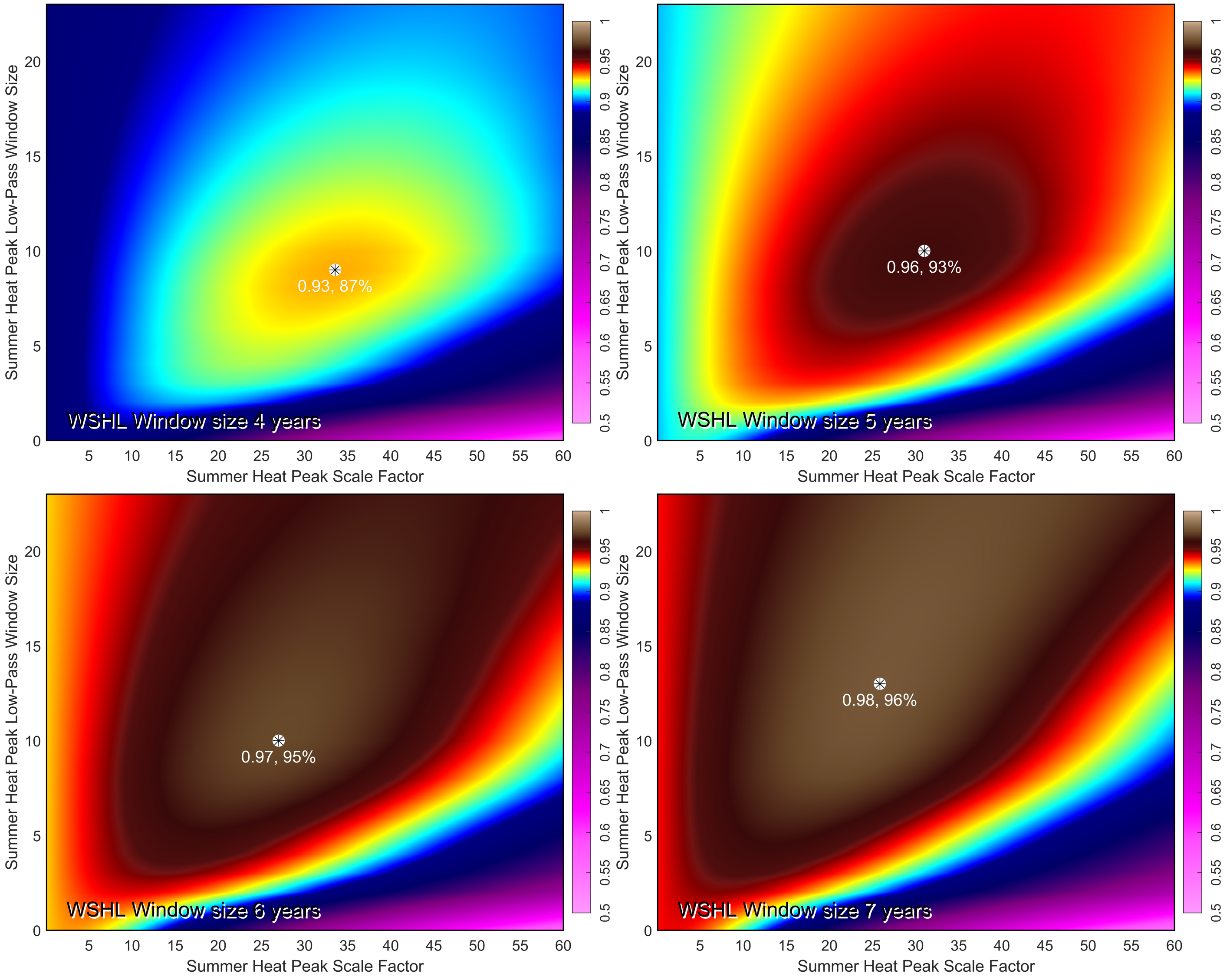


**Supplementary Figure 13.** Results of four steps of the multistep thermosteric height reconstruction optimization process performed through iterations over broad ranges of the tested variables (independent WSHL and SHP/SSHG low filer configurations, and the relative weights of the respective terms in the best fit).

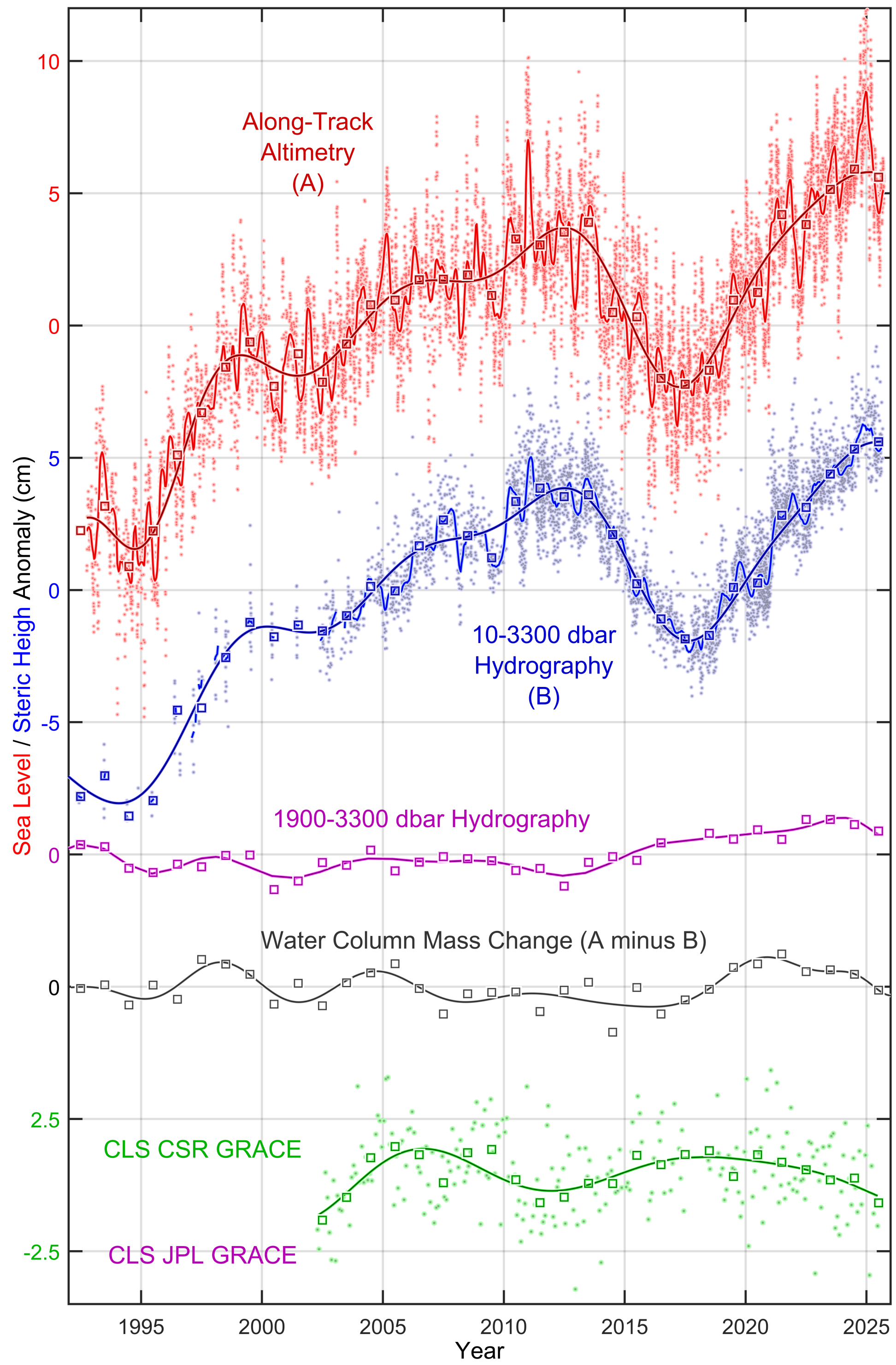


**Supplementary Figure 14.** 1992–2025 sea level budget variability in the central Labrador Sea. *Top-down* (cm): the along-track satellite altimetry-based sea level (*red*); full-depth (*blue*) and deep-layer (*purple*) steric heights; water column mass based on subtracting full-depth steric height from sea level (*grey/black*); and detrended CSR GRACE data (*green*). Outliers and respective regular (climatological) seasonal cycles have been removed from all original values. *Squares*, underlined with optimal polynomial fits, indicate the yearly averaged residuals. The summed irregular seasonal and low-frequency signals of (A) and (B) are shown with *lighter lines*.

**Table.** Labrador Sea hydrographic observations collected by the Bedford Institute of Oceanography in compliance with the Deep-Ocean Observation and Research Synthesis (DOORS) and World Ocean Circulation Experiment (WOCE) temperature, salinity and oxygen data quality requirements

| Cruise Name | Vessel | Project | Chief Scientist | Cruise Dates |
|---|---|---|---|---|
| DAW-90-012 | CCGS Dawson | WOCE | John Lazier | 2-Jul – 9-Jul, 1990 |
| HUD-91-007 | CCGS Hudson | WOCE | Ross Hendry | 24-Apr –24-May, 1991 |
| HUD-92-014 | CCGS Hudson | WOCE | John Lazier | 27-May – 15-Jun, 1992 |
| HUD-93-019 | CCGS Hudson | WOCE | John Lazier | 17-Jun – 28-Jun, 1993 |
| HUD-94-008 | CCGS Hudson | WOCE | John Lazier | 24-May – 12-Jun, 1994 |
| HUD-95-011 | CCGS Hudson | WOCE | John Lazier | 7-Jun – 5-Jul, 1995 |
| HUD-96-006 | CCGS Hudson | WOCE | John Lazier | 10-May – 2-Jun, 1996 |
| HUD-96-026 | CCGS Hudson | WOCE | Allyn Clarke | 15-Oct – 20-Nov, 1996 |
| HUD-97-009 | CCGS Hudson | WOCE | Allyn Clarke | 9-May – 12-Jun, 1997 |
| HUD-98-023 | CCGS Hudson | WOCE | John Lazier | 22-Jun – 10-Jul, 1998 |
| HUD-99-022 | CCGS Hudson | WOCE | Allyn Clarke | 27-Jun – 14-Jul, 1999 |
| HUD2000009 | CCGS Hudson | WOCE | Allyn Clarke | 20-May – 8-Jun, 2000 |
| HUD2001022 | CCGS Hudson | WOCE | Allyn Clarke | 30-May – 15-Jun, 2001 |
| HUD2002032 | CCGS Hudson | WOCE | Allyn Clarke | 23-Jun – 19-Jul, 2002 |
| HUD2002075 | CCGS Hudson | Climate | Erica Head | 29-Nov – 12-Dec, 2002 |
| HUD2003038 | CCGS Hudson | Climate | Allyn Clarke | 13-Jul – 4-Aug, 2003 |
| HUD2004016 | CCGS Hudson | Climate | Allyn Clarke | 14-May – 30-May, 2004 |
| HUD2005016 | CCGS Hudson | Climate | Allyn Clarke | 27-May – 7-Jun, 2005 |
| HUD2006019 | CCGS Hudson | DOORS | Ross Hendry | 24-May – 8-Jun, 2006 |
| HUD2007011 | CCGS Hudson | DOORS | Ross Hendry | 10-May – 29-May, 2007 |
| HUD2008009 | CCGS Hudson | DOORS | Glen Harrison | 20-May – 4-Jun, 2008 |
| HUD2009015 | CCGS Hudson | DOORS | Glen Harrison | 18-May – 1-Jun, 2009 |
| HUD2010014 | CCGS Hudson | DOORS | Glen Harrison | 13-May – 30-May, 2010 |
| HUD2011009 | CCGS Hudson | DOORS | Igor Yashayaev | 6-May – 29-May, 2011 |
| MLB2012001 | CCGS M.L. Black | DOORS | Igor Yashayaev | 25-Jun – 20-Jul, 2012 |
| HUD2013008 | CCGS Hudson | DOORS | Igor Yashayaev | 4-May – 28-May, 2013 |
| HUD2014007 | CCGS Hudson | DOORS | Igor Yashayaev | 2-May – 26-May, 2014 |
| HUD2015006 | CCGS Hudson | DOORS | Igor Yashayaev | 1-May – 26-May, 2015 |
| HUD2016006 | CCGS Hudson | DOORS | Igor Yashayaev | 30-Apr – 24-May, 2016 |
| HUD2018008 | CCGS Hudson | DOORS | Igor Yashayaev | 25-Apr – 20-May, 2018 |
| AMU2019001 | CCGS Amundsen | DOORS | Igor Yashayaev | 2-Jun – 19-June, 2019 |

## Supplementary Note 3. Responses to internal reviewers

**From:** Joël Chassé (Institut Maurice-Lamontagne, Québec, DFO)

I found your manuscript very interesting and you are trying to answer several questions in one publication! You certainly put a lot of effort in that work! Obviously, the novelty reside in the finding that the halosteric contribution to SLA has shifted from historically counterbalancing to recently reinforcing the thermosteric component, amplifying sea level rise in the Labrador Sea (LS). This is certainly worth publishing!!

You also quantified the deep-layer contributions to steric height which hadn't been done before (as far as I know).

My main comments are:

- You mostly attribute the 2023–2025 halosteric amplification to Arctic sea ice meltwater. While you give references, the evidence linking freshwater anomalies specifically to sea ice melt seems thin. Maybe that could be improved?

- Considering that the deep Argo coverage is quite sparse, I think that it would be worthwhile to discuss a bit more the robustness of the deep steric trends and how they are sensitive to data gaps and sampling errors.

- The method for reconstructing thermosteric variability from WSHL and SSHG/SHP is interesting but maybe a more physical justification for the choice of the specific time windows should be elaborated?

- The discussion of mass changes is intriguing and I remained on my appetite about it. But it seems that nothing else could really be done at this point (as you mentioned)? Could a comparison with ECCO mass budgets be quickly done?

- From your conclusions it feels like the LS is the unique region sensitive the halosteric–thermosteric reinforcement but maybe there should a paragraph about such possibility in other regions of deep convection (e.g., Irminger Sea or event Weddell Sea in the southern atmosphere)?

You will find attached the MS with some of my comments embedded in the PDF. I don't really have any major comment on the Supplementary Material section. We could have a call to discuss my comments if you judge it necessary.

I hope that helps a bit.

Again, great work!

Cheers,

Joël

**Point-to-point responses to Joël Chassé's general comments:**

Dear Joël,

Thank you so much for your comments. All and each of them are very helpful and highly appreciated!

Yes, you ask a right question - Where have the extra meters of freshwater come from?

The present paper is already too long for *Nature Communications* to provide the same argumentation as in the previous work (Intensification and shutdown of deep convection in the Labrador Sea were caused by changes in atmospheric and freshwater dynamics | Communications Earth & Environment) focusing on the processes, and there I considered Arctic sea ice loss, Beaufort Gyre freshwater release, and Greenland melt, and its Figure 9 from that paper shows why I vouched for the Arctic sea ice loss. We also show the seasonal Arctic sea ice loss in one of the last figures of the present paper. However, I totally agree with you that we need to strengthen this interpretation and point the reader not only to the previous paper, but to its updated and improved version that is now included in the present paper as Figure 9 (coincidentally). As part of the final revision, we included compelling evidence of the link.

There was something else that needed to be updated in the paper, and that is all done now – in 2024, the role of halosteric component reversed back to counterbalancing thermosteric – this is because the deeper layer started to get more salt as convection weakened (to record shallow in 2025) overriding the freshening of the upper layer that still continues as we speak. The explanation of the 2024 reversal to the expected counterbalancing regime is now included in the paper.

Another great question is the one concerning the quality of Deep Argo float data ... We did not say much in the previous version of the paper about the Deep Argo calibration and validation, and all technical steps I routinely perform on every float. This was my omission, because having two papers nearly stacked together, with one piggybacking the other, I naturally missed saying a word about the Deep Argo quality. We now address the readers to the Supplementary Information of the previously mentioned paper ("Intensification and shutdown …").

The method of thermosteric reconstruction. It is one of the coolest things of the work, in my humble opinion. Thank you for speaking so kindly of it.

The point of finding the right time scales for annual winter cooling and annual summer warming series (two separate cumulative heat flux series) was not in accepting any a priory hypothesis of actual scales. We extensively test all combinations of independent and related surface heat exchange metrics. The only hypothesis made here is that the two heat exchange processes, winter cooling and summer warming act differently ... The rest is achieved through computer programming — I wrote a code for successive testing of all possible combinations of the two time scales and the relative contributions of the cooling and warming series to the thermosteric height (*the result obtained here would perfectly work for the 10-2000 dbar heat content*). The best fit is then taken as the sought solution of the optimization problem (the *Supplementary Information* section has a figure showing just one slice of this optimization process and a commentary in *Supplementary Note 2*).

Why does the cumulative winter cooling impact the sea level on a shorter time scale than the summer warming? Could this be real? I strongly believe this is very real, and can think of a good explanation of this interesting fact — summer warming is a broader scale phenomenon with a spatial pattern of Atlantic Multidecadal Oscillation (AMO), while winter cooling is more in scale of what people like calling NAO (I use this very carefully as NAO is a very limited proxy of complex 4D weather pattern changes).

So, AMO is broader and has larger spatial and a longer time scale, whereas NAO in its subpolar core is more limited to the Icelandic Low variations (the Azores High contributes less to variability of NAO than the Icelandic Low). So, seeing shorter response to winter cooling and much longer to summer warming is kind of expected, if we think broadly about it, of course. Therefore, the result we get from the winter cooling and summer heating best fitting to thermosteric height (also heat content) make a perfect physical or climatological sense.

{*We do not make any statements about winter-based NAO vs summer-based AMP in the paper, as this interesting hypothesis would require a bit more testing, but the point we make there is clear, and the paper length I concern.*}

"Could a comparison with ECCO mass budgets be quickly done?"

Another amazing question, Joël!

I, personally, stay away from any gridded (e.g., EN4, ARMOR3D) or reanalysis (e.g., SODA, GLORYS, ECCO) products, I prefer either real ocean data or real ocean models, but my coauthor has given much of his PhD project to both ...

So, as soon as we saw this inconsistency between Altimetry minus Hydrography versus GRACE, we asked ourselves, what about ECCO? The answer turned out to be very simple – ECCO shows the same water column mass signal as GRACE! Naturally, this is what assimilation is about – the models do not change mass of the ocean, so the only way for mass data to be added is through assimilation, i.e., taking any existing data for a needed variable and plugging it into the data blending scheme. Since the mass of the ocean is not changed by a numerical ocean model, this is done by using the only dataset providing the oceanic mass, i.e., GRACE.

As a matter of fact, the importance of our present paper for everyone who relies on ECCO, and similar gridded products, is that one of the key ingredients has serious issues, like biases, drifts and other shortfalls ... Nobody really checked the satellite gravimetry for individual oceanic basins, and our papers sheds a sliver of light on the issue ... Note that this is not even a major point of the paper, just a bonus, making those who rely on the reanalyses aware of these issues.

"… maybe there should a paragraph about such possibility in other regions of deep convection"

Of course, we need to red-flag the issue about a full sign reversal in the halosteric-thermosteric relationship for other regions affected by anomalous inflow of freshwater, and this should be one of the follow-up investigations by whoever decides to take the lead on it!

**Responding to the points in Joël Chassé's annotated pdf file:**

"Yes, but the halosteric component started to rebalance the thermosteric component at the end of the time series. So, it should be mentioned that the 2025 high in SLA is mostly due to thermosteric component"

Yes, this happened between the first version of the paper we finished in the fall of 2024 and now. If we waited for a few more years, we would see something else, and the paper will grow in size even more ... I expanded the discussion about the most recent switch back to counterbalance in the end of the "Results" section.

Regarding the exclusive use of ship data, except for the missing survey year:

"Not sure that I understand what that means; where would the profiles come from if not from ship-based surveys?"

This was probably because we did not word it clear enough. In all analyses presented in the paper except Figure 7, we used real Argo and ship-based profiles, and from 2002 onward, Argo dominates.
But for the purposes of Figure 7, we wanted to be maximally consistent using the ship data except the two years for which only Argo observation were available. We need to rephrase the statement referring to the compilation presented in Figure 7 as all the other ones are done differently.

A good point about:

"I see some years (e.g. 1953-56) where both steric components would change in the same direction; worth mentioning?"

One of the anonymous reviewers pointed at the same things – possible short-term reversals in the past, and we addressed this concern not in the revised manuscript, but in the responses to reviewers. Since, we have two voices now raising the same question a short explanation will be added to the text.

We definitely saw the questioned reversals in the earlier years, and those kept me puzzled for years, because I manually check all T and S profiles from ships and Argo floats, and anything we see before 1962, even 1975, may be problematic in terms of quality. I trust the bottle data from the International Ice Patrol surveys if they show signals persisting for several years or profiles without big vertical inversions in salinity and density. Many questionable profiles I keep as in some years there is nothing else available.

Overall, a reversal in halosteric-thermosteric correlation can definitely happen for a short period of time, but we start with what  we recently saw as the longest and most evident halosteric-to-thermosteric reinforcement, and trying to find anything at least a half that long. Nothing showed as we know!

Very good comments altogether. Mostly pointing at our need to explain whatever we missed explaining in the present text and provide the missing links to the previous closely-related paper.

Thanks again,

Igor

## Supplementary References


Dickson, B., Yashayaev, I., Meincke, J., Turrell, B., Dye, S. & Holfort, J. Rapid freshening of the deep North Atlantic Ocean over the past four decades. *Nature* 416, 832–837 (2002).

Lazier, J., Hendry, R., Clarke, A., Yashayaev, I. & Rhines, P. Convection and restratification in the Labrador Sea, 1990–2000. *Deep Sea Research Part I: Oceanographic Research Papers* 49, 1819–1835 (2002).

Yashayaev, I. Hydrographic changes in the Labrador Sea, 1960–2005. *Progress in Oceanography* 73, 242–276 (2007).

Yashayaev, I. Intensification and shutdown of deep convection in the Labrador Sea were caused by changes in atmospheric and freshwater dynamics. *Commun Earth Environ* 5, 1–23 (2024).

Yashayaev, I., Bersch, M., & van Aken, H. M. Spreading of the Labrador Sea Water to the Irminger and Iceland basins. *Geophysical Research Letters* 34, L10602, (2007).

Yashayaev, I., Van Aken, H. M., Holliday, N. P. & Bersch, M. Transformation of the Labrador Sea Water in the subpolar North Atlantic. *Geophysical Research Letters* 34, 2007GL031812 (2007).
